# Regulation of Apoptosis by Enteroviruses

**DOI:** 10.3389/fmicb.2020.01145

**Published:** 2020-06-03

**Authors:** Yalan Lai, Mingshu Wang, Anchun Cheng, Sai Mao, Xumin Ou, Qiao Yang, Ying Wu, Renyong Jia, Mafeng Liu, Dekang Zhu, Shun Chen, Shaqiu Zhang, Xin-Xin Zhao, Juan Huang, Qun Gao, Yin Wang, Zhiwen Xu, Zhengli Chen, Ling Zhu, Qihui Luo, Yunya Liu, Yanling Yu, Ling Zhang, Bin Tian, Leichang Pan, Mujeeb Ur Rehman, Xiaoyue Chen

**Affiliations:** ^1^Institute of Preventive Veterinary Medicine, Sichuan Agricultural University, Chengdu, China; ^2^Key Laboratory of Animal Disease and Human Health of Sichuan Province, Sichuan Agricultural University, Chengdu, China; ^3^Avian Disease Research Center, College of Veterinary Medicine, Sichuan Agricultural University, Chengdu, China

**Keywords:** enterovirus, apoptotic pathway, regulation, balance, viral replication

## Abstract

Enterovirus infection can cause a variety of diseases and severely impair the health of humans, animals, poultry, and other organisms. To resist viral infection, host organisms clear infected cells and viruses via apoptosis. However, throughout their long-term competition with host cells, enteroviruses have evolved a series of mechanisms to regulate the balance of apoptosis in order to replicate and proliferate. In the early stage of infection, enteroviruses mainly inhibit apoptosis by regulating the PI3K/Akt pathway and the autophagy pathway and by impairing cell sensors, thereby delaying viral replication. In the late stage of infection, enteroviruses mainly regulate apoptotic pathways and the host translation process via various viral proteins, ultimately inducing apoptosis. This paper discusses the means by which these two phenomena are balanced in enteroviruses to produce virus-favoring conditions – in a temporal sequence or through competition with each other. This information is important for further elucidation of the relevant mechanisms of acute infection by enteroviruses and other members of the picornavirus family.

## Introduction

Enteroviruses (EVs) are nonencapsulated, single-stranded positive-sense RNA viruses that belong to the genus *Enterovirus* in the Picornaviridae family. Their complete genomic structure is VPg+5′UTR-[VP0-VP3-VP1/2A(2A1-2A2-2A3)-2B-2C/3A-3B-3C-3D]-3′UTR+Poly(A) (Yang et al., 2017). The main members of the genus *Enterovirus* are coxsackievirus B3 (CVB3), poliovirus (PV), and EV 71 (EV71). When an EV infects a host animal, it often causes various symptoms (Jubelt and Lipton, 2014). For example, PV can cause neurological symptoms such as aseptic meningitis and poliomyelitis (Korotkova et al., 2016). The symptoms of Coxsackie virus infection range from mild cold symptoms to severe myocarditis, pericarditis, meningitis, and pancreatitis (Muller et al., 2017; Qi and Xiong, 2017). EV71 infection is often characterized by hand, foot and mouth disease (Yee and Laa Poh, 2017). Thus, EV infection can severely endanger human health.

Host cells have developed a number of methods to protect against viral invasion, such as apoptosis. Apoptosis is a process of programmed cell death triggered by various stimuli, including viral infection. Since viruses infect host cells by binding to cell surface receptors and then replicate intracellularly, they use most of the host cell transcription and translation systems, and they generate various signals to activate the apoptotic pathway. The removal of infected cells by death receptor- or mitochondria-mediated apoptosis is a powerful innate immune mechanism for combating intracellular pathogens, since viral survival depends on cell survival until the pathogen completes its life cycle. The death of infected cells eliminates the environment required for pathogen replication (Harris and Coyne, 2014). When a virus infects cells, the cells initiate an apoptotic process, and viral replication is interrupted as the cells die. Therefore, the death of cells early in viral infection will severely limit production of the virus, thus reducing or eliminating the spread of the progeny virus in the host (Zheng et al., 2017). Because of these host mechanisms, viruses have developed various methods to inhibit apoptosis in order to promote their own replication (Heylbroeck et al., 2000; Yeganeh et al., 2018). On the other hand, the induction of apoptosis can also benefit viruses because apoptosis of virus-infected cells allows the virus to evade host immune recognition. Moreover, in the late stage of viral proliferation, the release of progeny virus can be promoted by triggering of apoptosis, which accelerates infection of nearby living cells (Zhou et al., 2017). Cell death has been reported to be extremely important for viral release (Sun et al., 2016), and the use of apoptosis inhibitors can suppress the release of virus (Deszcz et al., 2004). In summary, the proapoptotic and antiapoptotic effects of a virus on host cells play different roles during the life cycle of the virus. Therefore, the balance between proapoptotic and antiapoptotic processes is critical for viral replication (Harris and Coyne, 2014). With the advancement of research, accumulating evidence has indicated that EVs have evolved a series of mechanisms throughout their long-term competition with host cells that enable them to regulate apoptosis in order to replicate, proliferate and spread.

## Apoptotic Mechanism

In general, two different signaling cascades trigger apoptosis: the extrinsic apoptotic pathway and the intrinsic apoptotic pathway (Liu et al., 2004, 2019; Celine and Miquel, 2012; Guillermo et al., 2014). Caspase protein family members are key molecules in these pathways. Conditions that disrupt intracellular balance, such as mitochondrial dysfunction, calcium imbalance, and DNA mutation, can activate the intrinsic apoptotic pathway, which is mainly composed of and regulated by members of the multidomain B cell lymphoma 2 (Bcl-2) family (Shukla et al., 2017; Jin et al., 2018). This family includes the antiapoptotic molecules Bcl-2 and Bcl-XL (Perciavalle et al., 2012; Wen-Juan et al., 2012; Feng et al., 2018) and the proapoptotic molecules Bcl-2-related X protein (Bax) and BCL-2 antagonist (Bak) (Hsu et al., 1997; Mathai et al., 2005), among others. The intrinsic pathway is activated mainly by the interaction of Bcl-2-like protein 11 (BCL2L11, also known as Bim) or BH3-interacting domain death agonist (Bid) with Bax or Bak. Bax or Bak then undergoes a conformational change (Zhou et al., 2017) and forms heterodimers or homodimers that damage the mitochondrial membrane and reduce the mitochondrial membrane potential. Subsequently, the mitochondria release a protein that binds directly to inhibitor of apoptosis proteins (IAPs), second mitochondria-derived activator of caspases (SMAC), and cytochrome C (Cyt c) in the cytoplasm (Bossy-Wetzel et al., 2014; Kumari et al., 2017), initiating caspase-dependent apoptosis (Xinya et al., 2010). Cyt c binds to procaspase 9 (Shakeri et al., 2017) and the apoptotic protease activator Apaf-1 to form a complex, which cleaves procaspase 9 into caspase 9 and ultimately activates downstream caspase 3 (Liu et al., 2004; Wang G. et al., 2013). In addition, SMAC activates caspase 3 by inhibiting IAPs. Activated caspase-3 cleaves the DNA repair enzyme poly(ADP-ribose) polymerase (PARP), ultimately leading to apoptosis (Penny and Tyler, 2009). In addition, when the intrinsic apoptotic pathway is activated, mitochondria release apoptosis-inducing factor (AIF), a factor that induces caspase-independent apoptosis, in addition to SMAC and Cyt c. AIF is released from mitochondria and translocates to the nucleus, where it activates endogenous endonucleases to cleave nuclear DNA into 50 kb fragments (Qu et al., 2017).

Initiation of the extrinsic apoptotic pathway begins with the binding of death ligands to their corresponding receptors. Specifically, the death ligands TNF and Fas ligand (FasL) bind to their receptors, TNFR and Fas, and recruit the corresponding death domain (DD) proteins, TNFR-associated DD (TRADD) and FAS-associated DD (FADD) (Liu et al., 2004), respectively. These proteins then bind to procaspase 8 to form the death-inducing signaling complex (DISC) (Jiang et al., 2017), which activates caspase 8 and ultimately activates downstream caspase 3. However, mitochondrial apoptosis and death receptor-mediated apoptosis are not independent of each other. Activated caspase 8 can cleave Bid to form tBid with proapoptotic activity (Penny and Tyler, 2009), and tBid changes the mitochondrial membrane potential; this process activates the internal apoptotic pathway (Xiao-Min et al., 2004) and thus links the mitochondrial apoptosis with death receptor-mediated apoptosis (Chang et al., 2014; Zamzami et al., 2017).

## Counteraction of Apoptosis by EVs

To survive and replicate in cells, EVs have developed multiple strategies to block apoptotic processes. As early as 1995, some scholars discovered that PV can block cyclohexylamine-induced apoptosis and described the antiapoptotic functions of RNA viruses for the first time (Tolskaya et al., 1995). With further research, increasing numbers of EVs have been found to inhibit apoptosis.

### Cell Sensors and Apoptosis Inhibition

**FIGURE 1:**
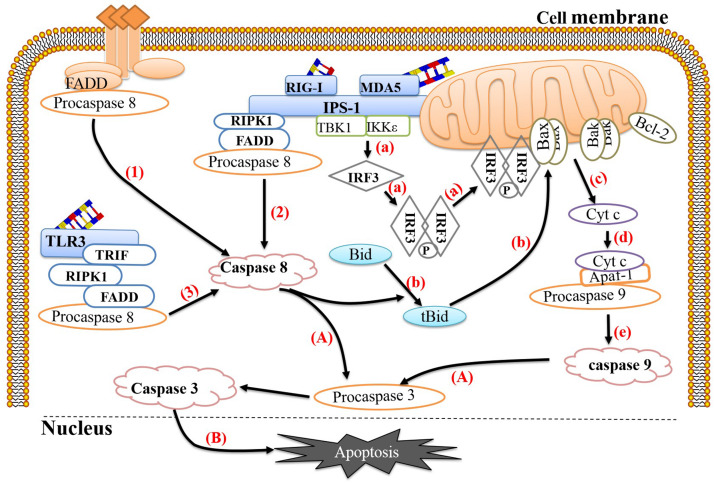
Link between pathogen recognition and apoptosis. (1) The death ligands TNF and FasL bind to their corresponding receptors, TNFR and Fas, and recruit TRADD and FADD, respectively, which then bind to procaspase 8 to form the death-inducing signaling complex (DISC) to activate caspase 8. (2) RIG-1 or MDA-5 recognizes viral dsRNA and recruits IPS-1. Activated IPS-1 recruits FADD and RIPK1 to form a complex and then recruits and activates procaspase 8. (3) TLR-3 recognizes viral dsRNA in the cytoplasm and exposes the TIR domain. The adaptor TRIF containing the TIR domain binds to RIPK. The C-terminus of RIPK1 has a death domain (DD), which can interact with FADD, after which FADD recruits and activates procaspase 8. (a) Activated IPS-1 can also recruit IKKε and TBK1, causing phosphorylation and dimerization of IRF-3. The IRF-3 homodimer can translocate to the mitochondrial membrane and form a complex with Bax. (b) Activated caspase 8 can cleave Bid to form tBid with proapoptotic activity, which can induce Bax and Bak to insert into the outer mitochondrial membrane. (c) Bax or Bak undergoes conformational changes and forms heterodimers or homodimers, thereby destroying the mitochondrial membrane and reducing the mitochondrial membrane potential. Subsequently, mitochondria release Cyt c into the cytoplasm. (d,e) Cyt c, procaspase 9 and Apaf-1 combine to form a complex that cleaves procaspase 9 into caspase 9. (A) Caspase 9 and caspase 8 cleave procaspase 3 into caspase 3. (B) Caspase 3 translocates to the nucleus and activates proteins, ultimately leading to apoptosis. The image content has been borrowed from other articles in the literature (Croft et al., 2017; Sun et al., 2019).

To protect against virus invasion, host cells can use multiple pattern recognition receptors (PRRs) to detect the presence of pathogens and then activate a series of antiviral responses to clear the pathogens. During the EV replication process, double-stranded RNA (dsRNA) is produced as an intermediate, which is recognized by corresponding PRRs, such as retinoic acid-induced gene 1 (RIG-1), melanoma differentiation-related gene 5 (MDA-5) and Toll-like receptor 3 (TLR-3). RIG-1 and MDA-5 activation occurs mainly during the later stages of infection and is coordinated with TLR-3 activation (Slater et al., 2010). Studies have shown that host cells can link the recognition of pathogens to the activation of apoptosis, thereby effectively preventing viral replication. RIG-1 mainly recognizes short dsRNA and single-stranded RNA containing 5′-triphosphate ends, while MDA-5 mainly recognizes long dsRNA (Lei et al., 2016). Both can recruit the adaptor protein IFN-β promoter stimulator 1 (IPS-1) (also known as Mavs or visa) through their intracellular RNA helicase activity (Kawai et al., 2005) and then recruit nuclear factor κB inhibitor kinase ε (IKKε) and TBK1, ultimately causing phosphorylation and dimerization of IRF-3 (Han et al., 2004; Croft et al., 2017). It is worth noting that the IRF-3 homodimer cannot only be transferred to the nucleus and induce the expression of the antiviral protein IFN-β but also be transferred to the mitochondrial membrane and form a complex with Bax, ultimately inducing apoptosis. In addition, activated IPS-1 can recruit FADD and receptor interaction serine (Ser)/threonine (Thr) kinase 1 (RIPK1) to form complexes and then recruit and activate pro-caspase 8 to induce apoptosis (Besch et al., 2009; Chattopadhyay and Sen, 2014). TLR-3 primarily recognizes dsRNA in the cytoplasm. When dsRNA binds to TLR-3, it can induce TLR-3 to form a homodimer, thereby exposing the Toll-IL-1 receptor (TIR) domain. TIR domain-containing adaptor-inducing β-interferon (TRIF) binds to TLR-3-activated IRF-3, TANK-binding kinase-1 (TBK-1), and nuclear factor κB (NF-κB), which subsequently activates IFN-β transcription (de Bouteiller et al., 2005; Sun et al., 2019). Studies have shown that overexpression of TRIF can induce apoptosis and signal transduction along the TRIF-RIPK1-FADD-caspase 8 axis (Estornes et al., 2012). In this process, RIPK1 plays an important role; RIPK1 contains a C-terminal RIP isotype interaction motif (RHIM) between its N-terminus and C-terminus that can interact with RHIM at the C-terminus of TRIF through homology (Kaiser and Offermann, 2005). The C-terminus of RIPK1 has a DD that can interact with FADD (Takeuchi and Akira, 2009) so that the apoptosis signal triggered by TLR-3 can be transmitted smoothly (Figure 1).

For EVs, the destruction of cell sensors not only allows the viruses to resist the cellular immune response but also prevents apoptosis from blocking the viral replication process. Numerous studies have shown that cell sensors can be cleaved by a variety of picornaviruses, suggesting the importance of this mechanism for the picornavirus life cycle. For example, cleavage of RIG-I has been found in PV-infected cells (Barral et al., 2009), and subsequent studies have revealed that transfection of the 3C protein alone or addition of the 3C protein *in vitro* can lead to RIG-I cleavage, indicating that the 3C protein can cleave RIG-I (Papon et al., 2009). The 3C protein of EV71 also has a similar effect. It can interact with the caspase recruitment domain at the N-terminus of RIG-I, thereby inhibiting RIG-1 from forming a complex with IPS-1, but it has no inhibitory activity on MDA5 (Lei et al., 2010). The 3C protein of CVB3 can also mediate the cleavage of RIG-I (Feng et al., 2014). Similarly, MDA5 in CVB3-, EV71-, and PV-infected cells undergoes protein degradation in a caspase- and proteasome-independent manner, and the 2A protein has been found to be involved in this process (Feng et al., 2014). The above results indicate that cleavage of RIG-I and MDA5 is a common strategy by which EVs resist the interferon response in infected cells. Since RIG-I and MDA5 play important roles in activating IPS-1 and recruiting FADD, RIPK1, and pro-caspase 8 and in activating IRF3 and Bax to form a complex, the cleavage of RIG-I and MDA5 by EVs may be related to the regulation of apoptosis.

Studies have shown that many EVs can also cleave IPS-1, which is a key protein in both the interferon response and apoptosis. Cleavage of IPS-1 can inactivate downstream signal transduction. In EV71-infected cells, the EV71 2A protein cleaves IPS-1 at the region between the proline-containing region and the transmembrane domain (Wang B. et al., 2013). In addition, the 3C protein of CVB3 can cleave IPS-1 in its proline-rich region, causing it to relocate from the mitochondrial membrane and eliminating its downstream signaling (Mukherjee et al., 2011). The 2A protein of CVB3 can also mediate IPS-1 cleavage (Feng et al., 2014). During PV infection, both the 3C and 2A proteins can cleave IPS-1 (Feng et al., 2014). When IPS-1 is cleaved, it can be released from mitochondria, which may cause the death receptor complex to be released from the mitochondrial anchor, thereby inhibiting apoptosis (Wang B. et al., 2013). Notably, EVs often use the same strategy to target specific host factors, but EVs use a variety of mechanisms when cleaving IPS-1.

In addition, phenomenon of TRIF cleavage consistent with that caused by 3C protein overexpression alone has been observed in CVB3-infected cells, confirming that the 3C protein is the enzyme responsible for cleavage of TRIF; the cleavage sites are located at the N-terminus and C-terminus of TRIF. The C-terminal RHIM motif of TRIF plays an important role in the transduction of apoptotic signals in the TRIF-RIPK1-FADD-caspase 8 axis. Therefore, cleavage of TRIF hinders not only the initiation of interferon transcription but also apoptosis (Mukherjee et al., 2011). Similar cleavage of TRIF has been observed in EV71-infected cells (Lei et al., 2011), indicating that this may be a common function of the EV 3C protein. However, whether TRIF can also prevent apoptosis after cleavage by EV71 has not been confirmed and needs further study (Table 1).

**TABLE 1:** Host proteins cleaved by enterovirus protease(s) during inhibition of apoptosis.

Host protein	Virus(es)	Protease(s)	References
RIG-I	PV	3C	Barral et al., 2009; Feng et al., 2014
	EV71	3C	Lei et al., 2010; Feng et al., 2014
	CVB3	3C	Feng et al., 2014
MDA5	PV	2A	Feng et al., 2014
	EV71	2A	Feng et al., 2014
	CVB3	2A	Feng et al., 2014
IPS-1	PV	3C 2A	Wang B. et al., 2013; Feng et al., 2014
	EV71	2A	Wang B. et al., 2013
	CVB3	3C 2A	Mukherjee et al., 2011
TRIF	EV71	3C	Lei et al., 2011
	CVB3	3C	Mukherjee et al., 2011

### PI3K/Akt Pathway and Apoptosis Inhibition

Regulation of the PI3K/Akt survival pathway is also an important mechanism by which EVs inhibit apoptosis. The PI3K/Akt survival pathway, which is composed mainly of PI3K and Akt, is a key antiapoptotic signaling pathway *in vivo*. PI3K activates Akt by phosphorylating Thr 308 and Ser 473 on Akt, and activated Akt phosphorylates various substrates to directly or indirectly to help cells survive. On the one hand, activated Akt can directly phosphorylate Ser 136 on Bad, Ser 184 on Bax, and Ser 196 on caspase 9, causing them to lose their ability to promote apoptosis and thereby effectively blocking apoptosis. On the other hand, activated Akt can affect cell survival by altering the activity of transcription factors such as NF-κB. Akt-mediated phosphorylation of NF-κB activates its transcription function, enabling NF-κB to enhance the expression of the antiapoptotic protein Bcl-xl and thereby promote cell survival. In addition, mammalian rapamycin (mTOR) is an important target downstream of Akt that can be activated by phosphorylation of Akt. It can control the translation of specific mRNAs, regulate protein synthesis and affect cell proliferation (Kim et al., 2001; Autret et al., 2008; Daxing et al., 2009; Shi et al., 2013; Li et al., 2018; Noorolyai et al., 2019).

**FIGURE 2:**
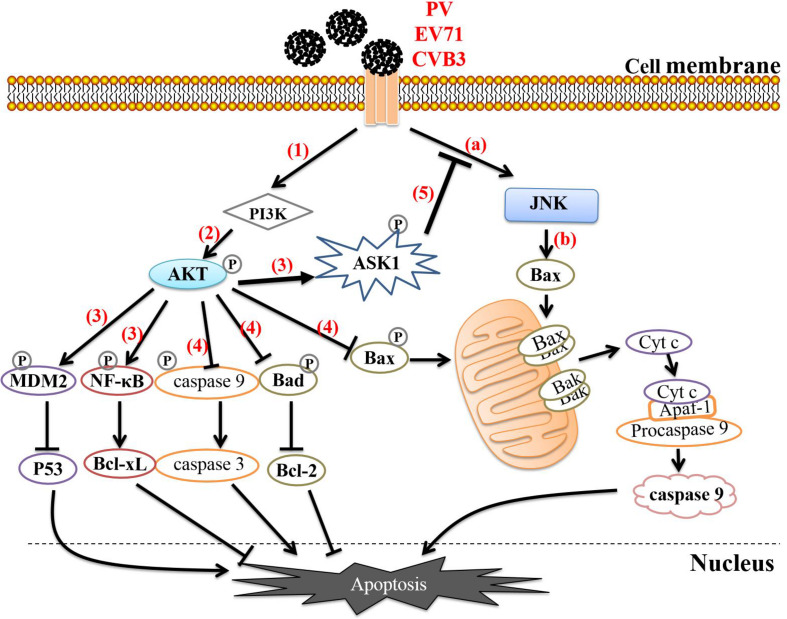
Enteroviruses regulate apoptosis through the PI3K/AKT pathway. (a) Enteroviruses interact with cellular receptors to induce JNK phosphorylation. (b) Activated JNK activates Bax-dependent apoptosis. (1) Enteroviruses interact with cell receptors to activate the PI3K/AKT pathway. (2) PI3K activates Akt by phosphorylating threonine (Thr) 308 and serine (Ser) 473 on Akt. (3) Activated Akt phosphorylates NF-κB, MDM2, and ASK1. Phosphorylation of NF-κB activates its transcription function, allowing it to enhance the expression of the antiapoptotic protein Bcl-xl. Phosphorylated MDM2 can inactivate or degrade P53, thereby blocking the P53-mediated proapoptotic transcription reaction. (4) Activated Akt directly phosphorylates Bad, Bax, and caspase 9, causing them to lose their ability to promote apoptosis and thereby effectively blocking apoptosis. (5) ASK1 is the upstream regulatory factor of JNK, and phosphorylated ASK1 negatively regulates the activation of JNK, thereby delaying activation of the proapoptotic protein Bax and effectively inhibiting apoptosis.

In the early stage of EV infection, the mitogen-activated protein kinase (MAPK)/ERK signaling pathway can be activated together with the PI3K/AKT pathway; this phenomenon has been observed in a variety of susceptible cells (Wong et al., 2005; Xie et al., 2016). The MAPK family can regulate a variety of cellular programs, including cell proliferation, the immune response, and apoptosis (Wang et al., 2012). PV interacts with cell receptors to induce phosphorylation of c-Jun amino terminal kinase (JNK) in the MAPK family. Activated JNK can enhance the activity of the transcription factor AP-1, thereby promoting the expression of proapoptotic proteins such as Bax, FasL, and TNF. It can also act on mitochondria through downstream signal transduction and promote Cyt c release. During PV infection of IMR5 cells, activation of JNK can activate Bax-dependent apoptosis, however, it also activates the PI3K/Akt pathway. Activated Akt can phosphorylate apoptotic signal-regulating kinase 1 (ASK1), an upstream regulatory factor of JNK, and thus negatively regulate JNK activation, thereby delaying the activation of the proapoptotic protein Bax and effectively attenuating apoptosis and prolonging cell survival (Autret et al., 2008). A similar phenomenon has also been observed in EV71- and CVB3-infected cells (Esfandiarei et al., 2004; Zhang et al., 2014b), suggesting that such a process may be widespread in EV-infected cells and may be related to EV-mediated prolongation of cell survival for completion of replication (Figure 2). Unfortunately, the specific details of the mechanisms underlying this process, such as the viral proteins that are involved, have not been thoroughly studied and require further exploration. Notably, EV71 can also simultaneously activate ERK1/2 and JNK1/2 of the MAPK family during infection of human intestinal epithelial cells, thereby downregulating TNF-α and FasL of the TNF family. This effectively inhibits apoptosis, extending the survival time of infected cells. When infected cells are treated with ERK1/2 and JNK1/2 inhibitors, the viral titers of EV71 decrease significantly, indicating that activation of these proteins does indeed enhance viral replication (Wang et al., 2015). However, the role of the PI3K/AKT pathway in this process has not been studied, so whether this pathway is activated and whether its activation is related to apoptosis inhibition remain unclear.

### Other Mechanisms of Apoptosis Inhibition

**FIGURE 3:**
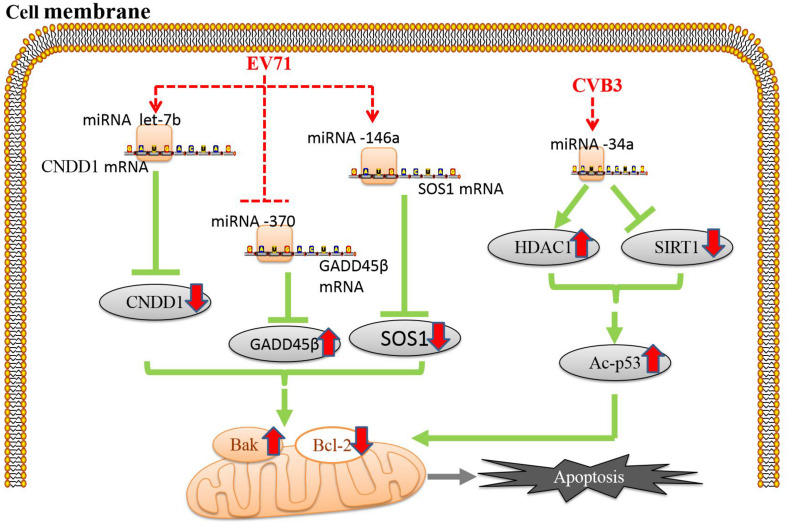
Enteroviruses regulate miRNAs to induce apoptosis. Apoptosis induced by enterovirus infection is associated with the regulation of microRNAs (miRNAs) (Chang et al., 2015; Jiang et al., 2019). The red dotted line indicates the site of enteroviral action, and the red solid arrows indicate the up- or downregulation of the expression of each protein.

EVs have also developed other ways to inhibit apoptosis. For example, after infecting cells, PV can increase the levels of the endoplasmic reticulum transmembrane protein Herp by regulating cAMP response element binding protein 3 (CREB3) in host cells. Herp is an important component of the endoplasmic reticulum-associated protein degradation pathway. It can reduce the Ca^2+^ concentration in the host cell cytoplasm by regulating the proteasome-mediated degradation of the Ca^2+^ release channels inositol 1,4,5-triphosphate receptor (IP3R) and ryanodine receptor (RyR), thereby inhibiting apoptosis (Delpeyroux et al., 2016). The 3A and 2B proteins of PV may clear the TNF receptor from the cell membrane after 4 h of infection by affecting the host cell endoplasmic reticulum-glial protein transport pathway, thus inhibiting TNF-mediated apoptosis early in infection (Neznanov et al., 2001). Under normal circumstances, autophagy is a cellular recycling mechanism and is generally considered a prosurvival response (Harris and Coyne, 2014). In the early stage of CVB3 infection of HeLa cells, apoptosis is inhibited via an increase in autophagosome formation (Xin et al., 2014). Similar inhibition of caspase-3 and apoptosis has been observed in the early stage of CVB3 infection in H9c2 cells (5–10 h after infection), and studies have shown that CVB3 enhances autophagy by activating calpain (Li et al., 2014; Figure 3).

## Induction of Apoptosis by EVs

Studies investigating EV-mediated inhibition of host cell apoptosis have also found that apoptosis can be induced during EV infection, which may aid in the release of the progeny virus and accelerate infection of surrounding living cells (Zhou et al., 2017). Various viral proteins play important roles in such virus-induced apoptosis.

### 3C Protein and Apoptosis

The 3C protein of picornavirus possesses the characteristic motif and activity of a chromoprotein-like cysteine protease. During the replication process, the only ORF of a viral small RNA is translated into a precursor polyprotein that is cleaved by the self-encoded proteases 2A, 3C, and 3CD into structural and nonstructural proteins with biological functional activity. The 3C protein has a wide range of restriction sites, so it can lyse certain cytokines in cells to regulate apoptosis. During EV71 infection, the EV71 3C protein can directly interact with caspase-8 and caspase-9, subsequently activating them and thereby indirectly upregulating caspase-3 activation (Song et al., 2018). The 3C protein of EV71 can also induce the cleavage of the DNA repair enzyme PARP by activating caspase, thereby triggering apoptosis (Li et al., 2002), and the 2A protein of EV71 has a similar effect (Kuo et al., 2002). During PV infection, a similar mechanism induces apoptosis by inducing PARP cleavage; this mechanism also involves both the 2A and 3C proteins. This effect of the PV 3C protein may be related to 3C-mediated cleavage of TATA binding protein that affects the normal progression of the cell life cycle (Clark et al., 1993; Barco et al., 2000; Calandria et al., 2004). However, whether the 3C protein of EV71 also causes PARP cleavage through this mechanism remains to be confirmed. These findings suggest that different EVs may share this strategy but implement it through slightly different mechanisms. The 3C protein of EV71 can also interact with the telomere-binding protein PinX1, which is subsequently cleaved at the specific site Q50-G51. Decreases in the expression of PinX1 can damage DNA in cells, triggering apoptosis (Li et al., 2016). Furthermore, the 3C protein of EV71 can cleave the host protein heterologous ribonucleic acid protein A1 (hnRNP A1), preventing the binding of hnRNP A1 to the internal ribosome entry site (IRES) of Apaf-1 and thereby promoting the transcription and translation of Apaf-1 (Li et al., 2019). In addition, Chau et al. found that the 3C and 2A proteins of CVB3 can lyse eukaryotic translation initiation factor 4GI (eIF4GI) in host cells, inhibiting cellular translation and ultimately causing cells to undergo morphological changes, shrinkage and other changes related to apoptosis (Chau et al., 2007). CVB3 also activates caspase-8 and caspase-9 by upregulating Bax expression via the 3C protein (Chau et al., 2007) and regulates the proapoptotic factors Bim, Bax, caspase-9 and caspase-3 in HeLa cells via mTOR, a downstream factor in the PI3K/Akt survival pathway (Xin et al., 2013; Table 2).

**TABLE 2:** Host proteins cleaved by enterovirus protease(s) during induction of apoptosis.

Host protein	Virus(es)	Protease(s)	References
PinX1	EV71	3C	Li et al., 2016
hnRNP A1	EV71	3C	Li et al., 2019
eIF4GI	PV	2A	Goldstaub et al., 2000
	EV71	2A	Kuo et al., 2002
	CVB3	3C 2A	Chau et al., 2007
DAP5	CVB3	2A	Hanson et al., 2015
Bid	CVB3	2A	Chau et al., 2007

### 2A Protein and Apoptosis

The structure and function of the picornavirus 2A protein vary widely among different species. This protein, as a proteolytic enzyme, plays an important role in virus-induced apoptosis. For example, studies have indicated that the 2A proteins of PV and EV71 can cleave eIF4GI in host cells, thereby inhibiting cap-dependent translation of host cell mRNA and preferentially inducing non-cap-dependent translation of apoptotic proteins (Goldstaub et al., 2000; Sh et al., 2002). However, during CVB3 infection, this mechanism is jointly mediated by the 2A and 3C proteins (Chau et al., 2007). In addition, the 2A protein of CVB3 cannot only cleave Bid to activate caspase 8 and caspase 9 (Chau et al., 2007) but also cleave death-associated protein 5 (DAP5), a structural homolog of eIF4G. DAP5 can regulate the transcription of apoptotic factors such as Bcl-2, p53, and XIAP driven by IRES elements and is cleaved by the 2A protein into N-terminal (DAP5-N) and C-terminal (DAP5-C) fragments. DAP5-N retains the ability to initiate IRES-driven p53 translation but lacks some of the effectiveness of DAP5 in initiating IRES-driven Bcl-2 translation. In addition, DAP5-N increases caspase-3 activation and can reduce Bcl-2 expression and enhance p53 translation. Thus, 2A protein signaling leads to increased apoptosis, thereby enhancing viral release (Hanson et al., 2015). Moreover, the 2A protein of EV71 may be a key inducer of apoptosis mediated by the apoptosis-related thioredoxin-interacting protein (TXNIP) (Yao et al., 2019; Table 2).

### 2B Protein and Apoptosis

Viroporin is a type of low-molecular weight virally encoded hydrophobic transmembrane protein with one to three hydrophobic transmembrane domains. These hydrophobic transmembrane regions can interact with the phospholipid bilayer as multimers to form transmembrane channels that change the permeability of the host cell membrane system (Gonzalez and Carrasco, 2003; Nieva et al., 2012). Viroporin thus destroys intracellular ion balance and regulates host cell apoptosis (Bagchi et al., 2010; Crawford et al., 2012). The 2B proteins of EVs such as PV, EV71, and CVB3 all have viroporin properties, indicating the importance of these properties for 2B proteins. Specifically, the viroporin property-mediated increases in membrane permeability induced by 2B proteins can alter Ca^2+^ homeostasis to regulate host cell apoptosis. PV increases Ca^2+^ flux from the endoplasmic reticulum into the cytoplasm by regulating IP3Rs and RyRs on the endoplasmic reticulum. When the Ca^2+^ concentration in the cytoplasm increases, the Ca^2+^ concentration in the mitochondria also increases. This regulatory effect causes mitochondrial dysfunction and triggers apoptosis in IMR5 neuroblastoma cells (Brisac et al., 2010; Son et al., 2014). This process may be related to the 2B protein of PV and its viroporin characteristics because increases in Ca^2+^ concentrations in the cytoplasm can be detected when the 2B protein is expressed alone in cells (Aldabe et al., 1997). It has also been found that the 2B protein colocalizes with endoplasmic reticulum and Golgi complexes and significantly reduces Ca^2+^ in the complexes (de Jong et al., 2008). Similarly, CVB3 can induce apoptosis of rat cardiomyocytes by regulating the apoptotic signal transduction process involved in Ca^2+^ flux (Li et al., 1999), and EV71 can activate calpain by increasing Ca^2+^ flux after EV71 infection, thereby inducing AIF-mediated caspase-independent apoptosis (Lu et al., 2013). However, the specific mechanisms by which these viruses regulate Ca^2+^ flux have not been explored. Given the fact that the 2B proteins of EVs such as PV, EV71, and CVB3 have viroporin properties, the mechanisms may be related to 2B proteins, but this possibility has yet to be confirmed. In addition, EVA71 has been reported to increase Cyt c release and activate caspase-3 by regulating the recruitment of the proapoptotic protein Bax and inducing a Bax conformational change (Han and Cong, 2017). This phenomenon has been found to be due to the mitochondrial localization of the EV71 2B protein through its C-terminus and the direct interaction of the 2B protein with Bax at the N-terminus, which triggers redistribution and activation of Bax (Wang and Li, 2019).

EV structural proteins are also involved in inducing apoptosis. The proapoptotic ability of the structural protein VP2 of CVB3 is related to its specific and independent interaction with amino acids 118–136 of the proapoptotic host cell protein Siva (Henke et al., 2001; Martin et al., 2004). The structural protein VP2 of PV can also bind to Siva and induce apoptosis (Henke et al., 2000).

### Other Mechanisms of Apoptosis Induction

PV can activate JNK by interacting with cellular receptors, thereby inducing Bax conformational change and redistribution from the cytoplasm to mitochondria (Autret et al., 2007). A similar phenomenon has also been observed in EV71- and CVB3-infected cells (Esfandiarei et al., 2004; Zhang et al., 2014b). EV71 also triggers cyclin-dependent protein kinase 5 (Cdk5) by activating the tyrosine kinase c-Abl, a key factor in neurotoxin signaling in the brain, to ultimately cause neuronal apoptosis (Chen et al., 2010).

Accumulating studies have shown that apoptosis induced by EV infection is associated with the regulation of microRNAs (miRNAs). miRNAs are a class of small, single-stranded noncoding RNAs widely found in eukaryotes that block the translation of their target mRNAs via posttranscriptional regulation, ultimately inhibiting target gene expression (Tang et al., 2016). In addition to engaging in the abovementioned mechanisms, CVB3 can target miRNA-34a in host cells to downregulate the histone deacetylase SIRT1, consequently increasing Bax expression, inhibiting Bcl-2 expression and inducing apoptosis (Jiang et al., 2019). Furthermore, p53-dependent apoptosis can be promoted by upregulation of the expression of the histone deacetylase HDAC1 via miRNA-34a (Zhou et al., 2015). EV71 can increase the expression of cyclin D (CCND1) by regulating the miRNA let-7b, thereby causing apoptosis of human neuroblastoma cells via cell cycle arrest (Du et al., 2015). EV71 can also regulate the apoptosis-related SOS1 and GADD45β proteins by upregulating miRNA-146a and downregulating miRNA-370, thereby reducing the expression of SOS1 and increasing the expression of GADD45β, respectively, which leads to downregulation of Bcl-2 expression and activation of caspase-3 and PARP (Chang et al., 2015; Figure 3).

## Mechanisms by Which EVs Balance Apoptosis

The above findings indicate that EV infection can both promote and inhibit apoptosis. With continuing research, scholars have gradually proposed a hypothesis to explain this contradictory phenomenon – the existence of a balance between viral promotion and inhibition of apoptosis. On the one hand, EVs can cause apoptosis by blocking the synthesis of host macromolecules to escape the host immune response and promote the release of progeny virus; on the other hand, viral proteins can exhibit antiapoptotic activity, thereby preventing premature death of host cells to prolong the replication time of the virus in living cells (Tolskaya et al., 1995). Timely inhibition and induction of apoptosis are considered two mechanisms that viruses use to achieve optimal replication (Croft et al., 2017).

### PI3K/Akt Pathway and Apoptotic Balance

How can this balance be achieved? According to the literature, different proteins of EVs may simultaneously exhibit antiapoptotic and proapoptotic functions. For example, when expressed in host cells, the 2A protein of PV can cleave eIF4GI and eIF4GII, thereby inhibiting the cap-dependent translation of host cell mRNA. However, the 3A protein of PV can neutralize the proapoptotic activity of the 2A protein by scavenging TNF receptors on the cell membrane; thus, PV exhibits antiapoptotic activity in the early stage of infection (the first 4 h) (Neznanov et al., 2001). In the later stages of infection, the direct effects of 3C, 2A and other viral proteins or the cell’s defense mechanism activates the caspase protein family through various apoptotic pathways, causing cells to undergo apoptosis. In addition, Mitra et al. (2003), Autret et al. (2008), Zhang et al. (2014b), and other research groups have found that PV and CVB3 proteins can activate the PI3K/Akt survival pathway by interacting with cellular receptors in the early stage of host cell infection and limiting JNK activation or activating the proapoptotic protein Bax. Initial delays in apoptosis allow a virus sufficient time to replicate inside a cell, but subsequent phosphorylation of JNK triggers apoptosis due to massive replication of the virus in the cell, thus allowing complete release of the virus (Autret et al., 2008). The literature reports that EV71 can inhibit apoptosis by activating AKT in the early stage of infection. However, in the late stage of infection, EV71 inhibits AKT phosphorylation by regulating the tumor suppressor gene RASSFs to promote apoptosis (Fengfeng et al., 2015). Therefore, the promotion and inhibition of apoptosis after EV infection may occur sequentially.

However, with increasing research, numerous studies have provided new explanations for the regulation of apoptosis by EVs. Apoptosis inhibition and promotion are not simply sequential; rather, they overlap. The apoptosis rates of infected HeLa cells have been reported to increase linearly within 1.5–2 h after PV infection, during which time replication of the viral genome is inhibited. However, in the next stage of viral reproduction (after 2 h of infection), caspase activation and apoptosis are inhibited. After initial entry of PV into a cell, the amount of viral protein produced by the translation of viral RNA, even if small, is sufficient to trigger apoptosis, which is a defense mechanism of the host cell itself. However, the accumulation of viral products with time (after 2 h of infection) prevents caspase activation (Agol et al., 2000). Notably, PV does not directly inactivate the caspase enzyme but rather prevents its activation, a function that may be related to the need for apoptosis induction in the late stage of infection. The above findings indicate that during EV infection, both inhibition and promotion of apoptosis may occur simultaneously in the same host cell, analogous to the two scales of Libra, but compete with each other for control over the host cell. This situation allows cells to exhibit either apoptosis or apoptosis inhibition at different stages. The infected cells thus outwardly exhibit sequential inhibition and promotion of apoptosis.

In addition, apoptosis induction during PV infection may be controlled by the balance among survival pathway activation at the early stage of infection, completion of the virus life cycle, and apoptosis at the late stage of infection. Notably, CREB3/Herp may be involved in the delicate balance between proapoptotic signals and antiapoptotic signals during PV infection. Upon infection, PV increases the expression of Herp by regulating CREB3 in host cells. Through this process, Herp expression peaks at 4 h after infection and then gradually decreases, while the Ca^2+^ concentration and apoptosis level increase beginning at 4 h (Delpeyroux et al., 2016). This finding may suggest that apoptotic processes temporally overlap with apoptosis-inhibiting processes. In addition, EV71 infection of HEK 293 cells and RD cells induces upregulation of hsa-miR-494-3p in cells, thereby increasing the expression of the targeted phosphatase gene PTEN. PTEN can activate the PI3K/Akt signaling pathway to inhibit early apoptosis and promote viral replication. Notably, Akt phosphorylation peaks at 0.5 h after infection and then gradually decreases (Zhao et al., 2018). In addition, during infection of HeLa cells, EV71 can induce the translocation of Src-associated mitotic protein (Sam68) from the nucleus to the cytoplasm. Sam68 can activate the PI3K/Akt signaling pathway by interacting with PI3K p85 to increase viral replication. During this process, Akt phosphorylation gradually increases, peaks at 1 h after EV71 infection, and then gradually decreases (Zhang et al., 2014a). This phenomenon is somewhat consistent with the findings discussed above.

### Autophagy and Apoptotic Balance

EVs can induce not only apoptosis but also autophagy during the infection process, and both play important roles in the life cycles of EVs. Autophagy is a catabolic pathway that is usually characterized by the formation of bilayer membrane vesicles (autophagosomes) that engulf cytoplasmic organelles and proteins and eventually fuse with lysosomes to reduce their lumen content (Klein and Jackson, 2011). Studies have shown that after EVs infect cells, autophagy and apoptosis can occur simultaneously, and the relationship between the two processes is very complex. For example, when CVB3 infects cells, the direct effects of viral proteins such as 3C and 2A or the cellular defense mechanism activates the caspase protein family through various apoptotic pathways to cause apoptosis, which is conducive to the release and transmission of the progeny virus. However, CVB3 also requires viral proteins such as 2BC and 3A to support autophagy through the formation of a replication complex that will continually replicate the viral genome to produce progeny virus. Therefore, CVB3 infection induces autophagy to promote replication. While the relationship between apoptosis and autophagy is complex, Shimizuet et al. have demonstrated that autophagy can act as a companion, antagonist or enabler of apoptosis depending on the cell type, stimulation and environment (Shimizu et al., 2004). When autophagy formation is reduced in CVB3-infected cells treated with the autophagy inhibitor 3-methyladenine (3-MA), caspase-3 activity is also reduced, and the rate of apoptosis is increased, however, the opposite effects are observed in cells treated with the autophagy inducer rapamycin. In contrast, CVB3 can induce the cleavage of autophagy-related proteins Beclin-1 and Atg5, and various caspase inhibitors can prevent the appearance of the Beclin-1 and Atg5 cleavage fragments. These results indicate that CVB3-induced apoptosis and autophagy are mutually antagonistic. However, it is interesting to note that CVB3-induced apoptosis is not consistent with the peak time of autophagy. The levels of the autophagy marker LC3-II increase from 3 to 9 h after CVB3 infection and then decrease, while the apoptosis rates of infected cells increase slowly from 3 to 24 h after infection (Xin et al., 2014). In addition, CVB3 can promote autophagosome formation by activating calpain. Analyses of dynamic curves of calpain and caspase 3 activation have yielded results similar to those described above. Calpain activity continuously increases until 10 h after CVB3 infection, while caspase-3 activity is significantly inhibited after infection. However, caspase-3 activity shows a downward trend before beginning to recover and stabilizing 10 h after infection (Li et al., 2014). This suggests that CVB3-infected cells may undergo a gradual transition from autophagy to apoptosis that may be related to the need for both in the CVB3 life cycle. CVB3 requires the use of autophagosomes during its replication phase. However, apoptosis can clear the virus to a certain extent, because the premature death of infected cells can interrupt viral replication, thus strongly impairing the reproduction of CVB3 (Zhang et al., 2002; Wong et al., 2008). Therefore, CVB3 uses apoptosis mostly to promote the release and spread of progeny virus in the late stage of infection.

**FIGURE 4:**
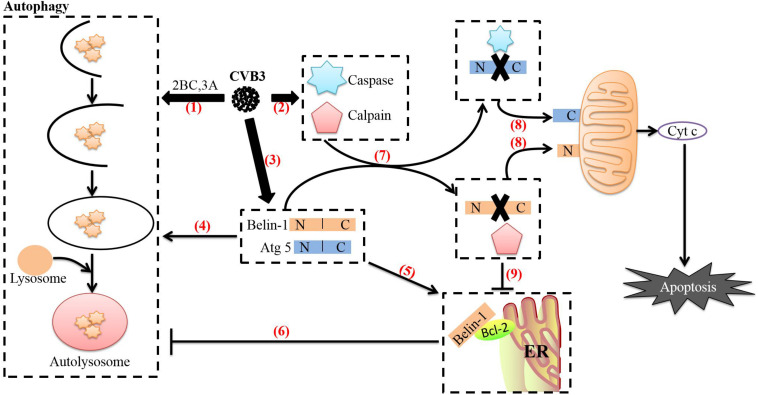
Relationship between autophagy and apoptosis induced by CVB3. (1) After CVB3 infects cells, it uses viral proteins (such as 2BC and 3A) to induce autophagy. (2) The direct actions of viral proteins (such as 3C and 2A) or the cell’s defense mechanisms then activate caspases and calpain through various pathways. (3) The transcription and translation of full-length ATG5 and beclin-1 in CVB3-infected cells continue to increase. (4) Beclin-1 aids in autophagosome initiation and autophagosome blockade, Atg5 aids in autophagosome extension, and Beclin-1 can stimulate high levels of autophagy. (5) Endoplasmic reticulum-localized Bcl-2 can form a complex with Beclin-1. (6) Upon forming a complex with Bcl-2, Beclin-1 loses its ability to induce autophagy. (7) Beclin-1 is cleaved by caspases, while Atg5 can be cleaved by calpains. (8) The C-terminal cleavage product of Beclin-1 is transferred to mitochondria and induces Cyt c release; the N-terminal cleavage product of Atg5 is also transferred to mitochondria and induces Cyt c release after binding to Bcl-xL. The release of cyt c into the cytoplasm leads to apoptosis. (9) Caspase-mediated destruction of the Beclin-1-Bcl-2 complex promotes autophagy. The image content has been adapted from other articles in the literature (Gordy and He, 2012; Jiang et al., 2014).

CVB3-mediated regulation of the transition between autophagy and apoptosis may be related to several mechanisms. First, as we have discussed previously, CVB3 can promote apoptosis through viral proteins such as 3C and 2A and ultimately activate caspase 8 (Li et al., 2011), caspase 9 (Rohn et al., 2011) and caspase 3 (Cho et al., 2009; Norman et al., 2010; Wirawan et al., 2010). The autophagy-related protein Beclin-1, which is required for CVB3 to induce autophagy, is a lytic substrate for caspase 8, caspase 9, and caspase 3. The C-terminal cleavage product of Beclin-1 can be transferred to mitochondria and induce Cyt c release (Wirawan et al., 2010). This process may mediate functional crosstalk between autophagy and apoptosis. Second, CVB3 can activate calpain through regulation of Ca^2+^ flux in cells via the 2B protein to increase the formation of autophagosomes. In CVB4-infected cells, calpain 1 and calpain 2 mediate the cleavage of the autophagy-related protein Atg5 (Yoon et al., 2008). The N-terminal cleavage product of Atg5 is then transferred to mitochondria, which induces Cyt c release after binding to Bcl-xL (Yousefi et al., 2006). In addition, caspases can inhibit autophagy by cleaving Atg5 (You et al., 2013). These processes may also be related to crosstalk between autophagy and apoptosis. Finally, interactions between Bcl-2 family proteins regulate not only apoptosis but also autophagy (Luo and Rubinsztein, 2010). CVB3 can regulate the expression of the proapoptotic protein Bax and the antiapoptotic protein Bcl-2 through miRNA-34a and Bax can attenuate Beclin-1-induced autophagy by mediating Beclin-1 cleavage (Luo and Rubinsztein, 2010). Endoplasmic reticulum-localized Bcl-2 can also inhibit Beclin-1-mediated autophagy by forming a complex with Beclin-1 (Pattingre et al., 2005; Maiuri et al., 2007; Strappazzon et al., 2011). Interestingly, caspase-mediated cleavage of Beclin-1 disrupts its interaction with Bcl-2. Although Beclin-1 C-terminal cleavage products and Bcl-2 maintain weak interactions under such conditions, the interaction between Bcl-2 and N-terminal cleavage products completely disappears. Notably, expression of N-terminal cleavage products of Beclin-1 can partially restore the formation of autophagosomes in Beclin-1 shRNA-expressing cells (Zhu et al., 2010), suggesting that caspase-mediated destruction of the Beclin-1-Bcl-2 complex may help promote autophagy. During the transformation from autophagy to apoptosis, the transcription and translation of full-length ATG5 and beclin-1 in CVB3-infected cells continues to increase, but overexpression of full-length beclin-1 can inhibit apoptosis and stimulate high-level autophagy (Cho et al., 2009). The balance between cleaved Beclin-1 and full-length Beclin-1 reflects the balance between apoptosis and autophagy (Cho et al., 2009; Wirawan et al., 2010; Zhu et al., 2010). Therefore, this strategy of CVB3 may be used to counteract the cleavage of caspase and calpain, prevent premature cell death, and thus ensure completion of CVB3 replication in autophagosomes (Figure 4).

Increasing numbers of reports have demonstrated that the replication of PV and EV71 is also related to the formation of autophagosomes. The viral proteins 2BC and 3A are necessary for the creation of a double-membrane vesicle environment for viral replication. However, CVB3 can also induce autophagosome accumulation by preventing autophagosome-lysosome fusion, which may be used to prevent the progeny virus from being degraded (Kemball et al., 2010). This phenomenon has not been found during PV and EV71 infection. In addition, although PV has been found to be able to induce both autophagy and apoptosis in separate studies (Barco et al., 2000; Corona Velazquez et al., 2018), there have been no studies on the relationship between the two processes at the same time; thus, further research is needed. It has been reported that EV71 can induce autophagy and apoptosis at the same time and that inhibition of the fusion phase of autophagosomes and lysosomes can promote apoptosis (Xi et al., 2013). In contrast, inhibition of EV71-induced apoptosis can promote the conversion of LC3-I to LC3-II. These mechanisms are similar to the mechanisms by which CVB3 regulates apoptosis and autophagy, suggesting that this strategy for balancing apoptosis in cells may be shared among EVs.

## Conclusion

**FIGURE 5:**
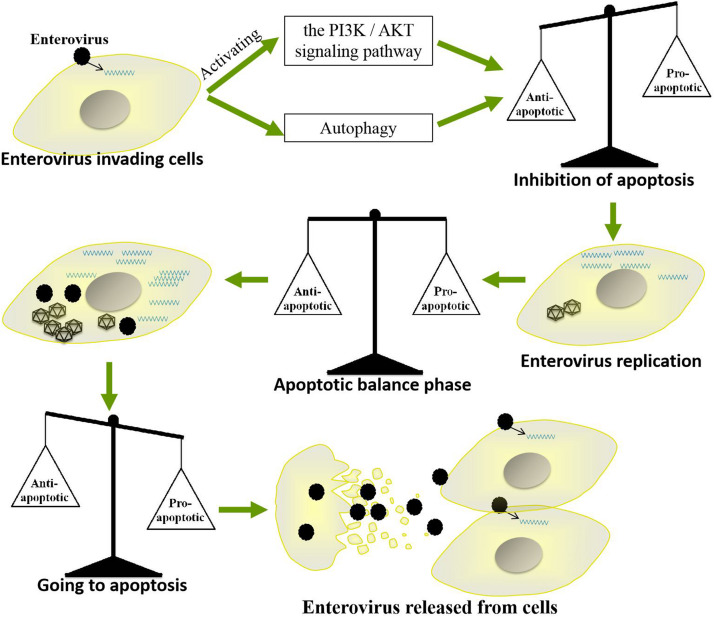
Mechanism by which enteroviruses balance apoptosis. Enteroviruses inhibit apoptosis in the early stages of infection to prolong their replication time but induce apoptosis in the late stage of infection to facilitate the release of progeny virus.

Apoptosis, which mainly eliminates viruses by killing infected cells, is a very important strategy by which cells resist viral infection. However, throughout their long-term competition with host cells, EVs have evolved a strategy by which they use apoptosis to promote their own replication and spread. For example, EVs inhibit apoptosis in the early stages of infection to prolong their replication time but induce apoptosis at the late stage of infection to facilitate the release of progeny viruses. Such precise regulation of host cell apoptosis enables EVs to replicate efficiently in cells and successfully spread to neighboring cells. However, the regulatory mechanisms by which these viruses balance the promotion and inhibition of apoptosis are very complicated (Figure 5). Further studies are needed to elucidate the specific mechanisms by which EVs regulate apoptosis and to determine whether as-yet-unidentified pathways are involved. Such studies will also be very important for further clarifying the pathogenic mechanisms of other small RNA viruses and for providing new insights for the development of antiviral drugs.

## Author Contributions

YLa and MW contributed to the design of the manuscript. SM, XO, QY, YWu, RJ, ML, DZ, SC, and QG provided ideas contributing to the conception of this manuscript. BT, SM, and XO helped to create the figures. AC modified the manuscript. All the authors reviewed the manuscript.

## Conflict of Interest

The authors declare that the research was conducted in the absence of any commercial or financial relationships that could be construed as a potential conflict of interest.
